# Skin shade and relationships: how colourism pits Black and mixed Black-White women against each other

**DOI:** 10.3389/fsoc.2024.1495048

**Published:** 2024-12-20

**Authors:** Aisha Phoenix, Nadia Craddock

**Affiliations:** ^1^Centre for Public Policy Research, School of Education, Communication & Society, Faculty of Social Science & Public Policy, King’s College London, London, United Kingdom; ^2^Centre for Appearance Research, School of Social Sciences, University of the West of England, Bristol, United Kingdom

**Keywords:** colourism, appearance ideals, heterosexual relationship market, dark-skin penalty, gendered differences, intersectionality, light-skin privilege, media influence

## Abstract

**Introduction:**

Colourism, prejudice where people are penalised the darker their skin and the further their features are from those associated with whiteness, occurs within and between racialised groups and can affect relationships. In this paper we examine the complex processes through which colourism positions Black and mixed Black-White women in contrasting positions in beauty and desirability hierarchies based on their skin shade.

**Method:**

Semi-structured interviews were conducted with 27 Black (*n* = 18) or mixed Black-White (*n* = 9) adults (18 women, 9 men) living in Britain. Data were analysed using reflexive thematic analysis.

**Results:**

Analysis generated four main themes. First, colourist and gendered appearance ideals promoted by mainstream media serve to devalue and thus harm Black people, particularly Black women with dark skin. Second, these ideals can affect Black and mixed Black-White women’s experiences of the heterosexual relationship market where women with light skin are desired and those with darker skin often overlooked. Third, colourist appearance ideals and colourist-induced inequities in the heterosexual relationship market affect relationships between Black and mixed Black-White women of different skin shades. Fourth, fostering Black self-acceptance, celebrating natural Black beauty, and creating spaces for dialogue between women of different skin shades are seen as ways to address some aspects of colourism and/or their affects.

**Discussion:**

Findings highlight how colourism shapes desirability in gendered ways and how this affects the lives of Black and mixed Black-White women, influencing within-group social dynamics and relationships.

## 1 Introduction

Colourism refers to prejudice in which people are penalised the darker their skin is and the further their features are from those associated with whiteness. Intrinsically related to, and yet distinct from racism (i.e., prejudice and discrimination based on racialised), colourism occurs both within and across racialised groups (Dixon and Telles, 2017). When colourism is experienced from a different racialised group, it can intensify experiences of racism, such that those with darker skin are more likely to experience racialised discrimination (Brown et al., 2023; Hunter, 2007). In this way, colourism and racism operate together (Hunter, 2007). However, within a racialised group, the prejudice operates in the absence of racism as a skin shade (and features) hierarchy in favour of those with lighter skin and phenotypical features that are associated with whiteness.

Colourism has a widespread impact on the lives of People of Colour^1^ globally and can affect many different aspects of an individual’s life. While people with light skin often benefit from light-skin privilege, those with dark skin experience penalties in numerous institutional settings, including in education, health and the criminal justice system (Crutchfield et al., 2022; Keyes et al., 2020; Monk, 2015; Monk, 2021). Colourism also can affect experiences in the relationship market, particularly for women (Hill, 2002; Hunter, 2021). As a racialised and gendered phenomenon, colourism can affect people differently according to their ethnicity and gender (Hunter, 2007; Dixon and Telles, 2017). Consequently, it is valuable to take an intersectional approach that is attentive to how colourism is perpetuated, reproduced and experienced at the individual, interpersonal, and systemic level in relation to the interplay of different personal characteristics, such as skin shade, ethnicity and gender. In this article, we focus on the experiences of Black and mixed Black-White women in recognition of the ways in which colourism compounds experiences of anti-Black racism.^2^

Colourism is pervasive and it is sustained and reproduced in multiple ways through powerful sociocultural influences including family, peers, and the media (Phoenix and Craddock, 2024a; Wilder and Cain, 2011; Phoenix and Craddock, 2022). In this article, we focus on how the media can serve to popularise, normalise, and perpetuate colourist ideas through the ways in which it both depicts (Harper and Choma, 2019) and excludes minoritised ethnic people, and particularly women with dark skin (Mitchell, 2020). The media, including film, TV, advertising and print media (e.g., newspapers and magazines), plays a potent role in contributing to wider colourist attitudes that can be internalised by individuals and reinforced by family and peers.

Operating alongside racism, colourism informs dominant media-sustained appearance ideals for women, which serve to privilege whiteness and light skin as attractive and desirable (Silvestrini, 2020; Hunter, 2021). For example, Keigan et al. (2024, p. 2) argue that “Eurocentric physical characteristics,” such as thinness, whiteness and long, straight hair are portrayed as the epitome of beauty” in the media. By positioning women with White or light skin as beautiful and desirable, women with dark skin are implicitly positioned as lacking in both beauty and desirability by the media and society more broadly (Collins, 2000; Hunter, 2021). For example, Collins (2000, p. 69) argues that “controlling images,” which are designed to make forms of social injustice, such as racism, sexism, and colourism, “appear to be natural, normal, and inevitable parts of everyday life,” construct Black women as “ugly and unfeminine” (p. 27).

Due to “patriarchal patterns of desire” (hooks, 2003), women and men’s experiences of colourism can differ, as women are typically judged on their looks more than men are, subjected to more societal appearance pressure, and held to different appearance ideals where light skin is consistently privileged (Hill, 2002; Phoenix, 2014). Additionally, as dark skin is often associated with masculinity and virility, Black men, unlike Black women, can benefit from having darker skin in the context of societal appearance ideals and romantic relationships (Hill, 2002; Phoenix and Craddock, 2022).

Colourism can inform heterosexual men of colour’s choice of romantic partners, and their descriptions of the physical attributes that make women attractive (hooks, 2003; Phoenix and Craddock, 2022). For example, Hunter (2002, p. 189) found that when it came to the marriage market, Black women in the USA who had light skin “had a clear advantage” over those with darker skin and were more likely to marry men of high-status. Experimental research from the US showed that Black men rated the same set of advertising images featuring Black women as significantly more attractive when their skin was digitally lightened compared with when their skin was digitally darkened (Watson et al., 2010). Black men in Phoenix and Craddock’s (2022) UK study explained that one reason why some Black men prefer women with lighter skin is due to the elevated social status they can gain from having a partner with light-skin privilege.

### 1.1 Colourism in the UK

Colourism is under-researched in the UK. However, a few studies have been published in the past few years that give some insights into how the prejudice operates in the UK. For example, in a cross-sectional, mixed ethnicity study on colourism, Craddock et al. (2023) found that compared with people from other minoritised ethnic groups, Black people reported greater experiences of colourism. Further, those with darker skin shades reported greater experiences of colourism. In turn, colourism was associated with negative wellbeing outcomes even when racism was included in the model, thus highlighting the unique role of colourism. Notably, qualitative work conducted in the UK that has explored the effects of colourism and racism has shown that in predominantly White areas, people of mixed ethnic backgrounds and Black people with light skin may be subjected to anti-black racism and may not experience the privileges they may otherwise experience in more diverse contexts (Phoenix and Craddock, 2024b; Spratt, 2024).

### 1.2 The present work

In this paper, we analyse narratives from Black and mixed Black-White women and men in an exploration of how skin shade affects Black and mixed Black-White women’s social positioning in the context of romantic relationships. Specifically, we take an intersectional approach focusing on ethnicity, gender and skin shade to explore the complex processes through which colourism positions Black or mixed Black-White women who have dark or light skin in hierarchies of beauty and desirability (see also Phoenix and Craddock, 2022; Hunter, 2021). We consider the implications of this in terms of both how women are positioned in the heterosexual relationship market, and how it affects relationships between women with dark and light skin.

Our work is situated in Britain (which includes England, Scotland, and Wales). Britain is majority White ranging from 81.7% White in England and Wales, to 87.1% White “Scottish” in Scotland (Office for National Statistics (ONS), 2022; National Statistics, 2024). The Black population (categorised as Black, Black British, Caribbean or African) is 4.0% in England and Wales and 1.0% in Scotland, and the mixed Black-White population is 1.1% in England and Wales.^3^ Britain has a long history of racism, including in its institutions (Afzal, 2022; Casey, 2023; Macpherson, 1999). Accordingly, our work examines colourism against a backdrop of racism in Britain.

## 2 Method

### 2.1 Participants

Participants were 27 adults (18 women, 9 men) racialised as Black (*n* = 18) or mixed Black-White (*n* = 9) living in Britain.^4^ The mean age was 35.8 years, ranging from 19 to 60 years old. Six participants (five women and one man) were full-time students (undergraduate or postgraduate), and one woman was a stay-at-home parent at the time of interview. The remaining participants worked in a variety of occupations in sectors including government, healthcare, media, and transport. All participants identified as heterosexual, except for one woman who identified as pansexual. Demographic data pertaining to social class or social economic status was not collected. Participant demographics, self-reported skin shade, and allocated pseudonyms are detailed in Table 1.

**Table 1:** Participant demographics.

Pseudonym	Gender	Age (years)	Generation cohort^a^	Ethnicity	Skin shade^b^
Ella	Woman	18–25	Generation Z	Mixed: Black-White	
Sofia	Woman	18–25	Generation Z	Black British	
Alice	Woman	18–25	Millennial	Mixed: Black-White	
Catherine	Woman	26–35	Millennial	Black British	
Chloe	Woman	26–35	Millennial	Mixed: Black-White	
Destiny	Woman	26–35	Millennial	Black British	
Erika	Woman	26–35	Millennial	Black British	
Jamila	Woman	26–35	Millennial	Black British	
Jennifer	Woman	18–25	Millennial	Mixed: Black-White	
Kelly	Woman	26–35	Millennial	Black British	
Sienna	Woman	18–25	Millennial	Mixed: Black-White	
Yemi	Woman	26–35	Millennial	Black British	
Clare	Woman	46–55	Generation X	Black British	
Grace	Woman	36–45	Generation X	Mixed: Black-White	
Portia	Woman	46–55	Generation X	Black British	
Viv	Woman	46–55	Generation X	Mixed: Black-White	
Dawn	Woman	56–65	Boomer	Black British	
Josephine	Woman	46–55	Boomer	Mixed: Black-White	
Terrence	Man	18–25	Generation Z	Black British	
Bilal	Man	26–35	Millennial	Black British	
Ekow	Man	26–35	Millennial	Black British	
Henry	Man	26–35	Millennial	Mixed: Black-White	
Isaiah	Man	26–35	Millennial	Black British	
Malakai	Man	26–35	Millennial	Black British	
Andrew	Man	36–45	Generation X	Black British	
Michael	Man	36–45	Generation X	Black British	
Gregory	Man	56–65	Boomer	Black British	

a Generational cohorts were based on participants’ ages at the time of their interview (2019) in line with Pew Research Center’s (2019) classification.

b The colour swatch participants selected from a purpose-built skin shade chart that best matched their skin shade.

### 2.2 Materials

#### 2.2.1 Skin shade chart

We created a purpose-built skin shade chart using 30 colours from Pantone® SkinTone™ Guide to use as an ice-breaker, and to capture participants’ skin shade as they saw it. The Pantone® SkinTone™ Guide is designed to be representative of the full spectrum of human skin types and in 2019 contained 110 SkinTone™ Guide shades. We selected 30 shades that we felt adequately represented minoritised ethnic people from different racialised groups and that were sufficiently distinct when printed on a home printer. We felt that 30 shades gave enough range without being overwhelming to look at in a brief exchange at the start of the interview.

#### 2.2.2 Interview guide

We followed a semi-structured interview schedule that we designed for the scoping research project. The semi-structured interview format provided flexibility, allowing the conversation between the interviewer and participant to delve into areas that felt most relevant to the participants, in such a way that data were co-constructed. Interview questions were intentionally broad to gain initial insights into the topic of colourism in Britain. For example, questions included: *How do you think others view your skin shade? Do you feel pressure to change the shade of your skin? Have you ever received preferential treatment because of the shade of your skin? Have you ever been discriminated against or treated badly because of the shade of your skin?*

### 2.3 Procedure

Ethical approval for a broad, scoping qualitative research project on colourism and skin shade in the UK was granted by the Research Ethics Committee at SOAS, University of London. Study advertisements were shared on social media inviting minoritised ethnic people (aged 18 and over) living in the UK to take part in a 60-min interview on their experiences related to their skin shade. Snowball sampling was also used. Interested individuals were sent a study information sheet and consent form. There were no incentives for participation.

Semi-structured interviews took place in person or via video conferencing software. The first author conducted 18 interviews, and the second author conducted nine. Demographic information was collected before formally starting the interview, with participants either completing a short questionnaire or answering demographic questions verbally. Then, we asked participants to indicate the colour that was the closest match to their skin shade on the chart before we proceeded with the interview, using our schedule.

Interviews were audio recorded with participants’ consent and were then transcribed by either a professional transcription service, freelance transcribers, or one of the study authors. To protect participants’ privacy, pseudonyms were used and identifying information was removed from participant accounts. Audio recordings were deleted once transcripts were checked by one of the study authors.

### 2.4 Data analysis

The first author led the data analysis using reflexive thematic analysis (Braun and Clarke, 2013, 2021). This approach was selected as it is a theoretically flexible approach, suitable for under-researched topics.

Analysis was informed by both social constructivism and intersectionality. Socio-constructivism posits that reality is socially constructed through interactions and shared understandings among individuals (Berger and Luckmann, 1966). There is no single, objective reality independent of human perception and social context. Reality is seen as constructed through language, culture, and social practices. Socio-constructivism emphasises the role of human agency in creating and interpreting the world (Burr, 2003).

Adding an intersectionality framework allowed us to understand the nuanced and multifaceted experiences of participants in relation to their personal characteristics, with a particular focus on gender, minoritised ethnic group and skin shade. Intersectionality, a concept coined by Crenshaw (1989), emphasises the interconnectedness of social identities such as “race,” gender, age, class, and sexuality, and how these intersections create unique modes of discrimination and privilege. By acknowledging that individuals’ experiences cannot be fully understood through single-axis analyses, this approach allows for a more comprehensive exploration of how various forms of inequality and identity overlap and interact (Collins and Bilge, 2016). Further, intersectionality provides a lens through which to examine the complexity of participants’ lived experiences and the structural contexts that shape them (Cho et al., 2013). By drawing on this framework, the study aims to capture the diverse and intersecting factors that influence participants’ understandings and experiences of colourism in the context of desire and relationships. However, it should be noted that our intersectional analyses are most attentive to gender, ethnicity and skin shade based on a predominantly heterosexual sample. Interpretation based on age and social class were beyond the scope of the present work as our sample was not large enough to draw meaningful comparisons and interpretation.

The six-stage guidance for reflexive thematic analysis detailed by Braun and Clarke (2013) was followed: (i) data familiarization, (ii) generating initial codes, (iii) generating themes, (iv) reviewing potential themes, (v) defining and naming themes, and (vi) writing up the results. Codes were generated in a two-step process. First, interviews were coded openly, guided by participants’ meanings. Then, a second round of deductive analysis was conducted, drawing on existing knowledge and understandings of colourism. The authors had several discussions about the data and candidate themes. The second author also provided feedback and revisions on the defining and naming of themes and the write up.

### 2.5 Positionality and reflexivity

Both authors are minoritised ethnic women. The first author is an African Caribbean sociologist. The second author has an educational background in psychology and is of mixed ethnicity, with one Indian and one White parent. We both have experience of using qualitative methods and interviewing individuals on sensitive and personal topics.

We did not seek to match participants’ broad racialised group when we assigned an interviewer as we were interested in exploring participants’ experiences as both “insiders” and “outsiders.” As an African Caribbean woman with dark or medium-dark skin, the first author had an insider status when interviewing Black women, some of whom remarked on how easy it was to talk to her. However, it may have been more awkward for mixed Black-White women to discuss the tensions they felt with Black women in conversation with a Black woman researcher. In contrast, the second author occupied insider status with participants of mixed ethnicity who all similarly had one White parent, which fostered some shared understanding in racialised dynamics and feelings of belonging. The second author’s outsider status in terms of racialised group may have been useful as it may have prompted more explanation and description, however, it may have limited the extent to which participants’ felt comfortable sharing.

As two women, we could not match interviewer-participant by gender for the men in our study. Prior to data collection, we wondered if men may be reticent about openly expressing their feelings on a sensitive topic that includes gendered dynamics. In fact, we found that men were forthcoming in their narratives. However, we do not know how they would have responded had they been speaking with a man, and particularly with one from the same racialised group as themselves.

## 3 Results

Our results are presented in four sections that consider the implications of different aspects of colourism for Black women with dark skin and Black and mixed Black-White women with light skin. The first explores the effect of colourist appearance ideals and colourism in the media. The second examines the impact of internalised colourism informing heterosexual relationship choices. The third analyses how sexual colourism causes competition and tensions between women. The fourth section considers Black participants’ small acts of resistance when confronted with colourist, societal appearance ideals.

### 3.1 “The media portrays an image that tells Black people … you are not beautiful”: mainstream media promoting colourist appearance ideals

In this first section, we begin by exploring women’s perceptions of how racist and colourist “European standards of beauty” promoted by the media contribute to constructions of Black women as inferior and unattractive. We then examine narratives that argue that women with very dark skin and natural hair are underrepresented or negatively represented in the media, which can have adverse psychological effects and contribute to Black women feeling undesirable. Next, we analyse narratives from Black men who argue that the media promotes the interests of White people, portraying Black women in negative ways, which can lead them to feel unattractive.

Many participants pointed to the media to explain why Black women with dark skin and Afro hair are positioned at the bottom of what Hunter (2023) refers to as the ‘beauty queue.’ The term describes how women are ranked by beauty (defined in large part by racialisation and skin shade) in marriage and dating markets, where “beauty” operates as a form of racialised social or symbolic capital. For instance, Kelly, a Millennial who described her skin shade as “Galaxy milk chocolate,” said:

*[P]eople tend to be like, ‘Oh, Black women are ugly’ or ‘Black women cannot grow their hair’ or ‘our hair is dry and picky’ or if you do not have a particular texture hair or whatever, you are not seen as beautiful as someone else…What else? Or, yeah, like we are just less than, because I mean look at everything in terms of the media around us, just like, you know, everything’s very European standards of beauty and all that kind of stuff.”*

Kelly suggested that dominant perceptions of Black women are that they are inferior (“less than”) and unattractive with unpleasant hair. Consistent with scholars such as Keigan et al. (2024), Kelly linked this to racist and colourist “European standards of beauty” in the media that privilege White or light skin and straight European hair.

In keeping with findings from a content analysis in the US, which found that Black women with dark skin were often excluded in media advertising (Mitchell, 2020), Alice, another Millennial, who had lighter skin, highlighted the lack of representation of Black and Asian women with “really dark” skin in the media.

*We’re continuously seeing the same type of Black woman or Asian woman [in the media]. It makes you think that’s the only way that [a] person should look, whereas we see a lot more different types of White women or– so you immediately think there’s one specifically attractive Black woman and if you do not fit that mould– or Asian woman or whatever then, I think it does, it does feed into your head. […] You see a lot fewer really dark women, I think, in magazine and films, and I also think you see a lot less natural hair.*

Her comment that “if you do not fit that mould … it does feed into your head,” suggests that there are psychological consequences of being excluded from the limited racialised constructions of beauty in the media. In addition to a lack of women with very dark skin and natural hair in the media, Alice said there is a “very narrow idea of what’s normal and what’s good,” indicating how morality is implicitly tied into media appearance ideals; that dark skin and Afro hair is neither “normal” nor “good.” This is linked to what Hunter (2021, p. 88) described as “a colour-based halo effect”: that light skin is not only associated with beauty but also virtue and other positive qualities.

It was not just Millennial women who spoke about the negative impact of racialised and colourist appearance ideals for women. Portia, who belonged to Generation X and described her skin as “beautiful dusky brown,” said she used to feel “too dark, not nice” and highlighted the media as one of the sources of that negative self-perception. She said:

*[W]e’re all brainwashed with all this stuff… daily from the media, from work, from society, you know everywhere you turn really, the news, everywhere. There’re so many messages that tell you that, you know, you are the other. And you are not, you know, the preferred option.*

Her narrative suggests that while the media plays a role in indoctrinating people with colourist ideas, colourist messaging is ubiquitous (“everywhere”). Portia also highlighted a gender disparity in the representation of Black people in the media in relation to skin shade. She said that “[d]arker-skin Black men […] they are viewed as attractive, whereas, I think for the most part, dark-skinned Black women probably still are not, if you look at the television and/or that kind of industry.”

Black men in our study also discussed the negative impact of racist and colourist gendered appearance ideals on Black people, and Black women in particular. For example, Ekow, a Millennial, said that the media can also have a damaging effect on Black people’s self-esteem through the images it conveys about beauty. He stated:

*[T]he media portrays an image that tells Black people, especially Black women, that you are not beautiful. So, unless you have a strong sense of self, or you are growing up around a community of people, that reinforce your own sense of identity, and your own sense of beauty, you can fall into the view that you are not beautiful, and you are not worthy.*

What he described is what Collins (2000, p. 69) terms “controlling images.” Collins (2000, p. 27) argues that some Black women internalise controlling images that construct them as “ugly and unfeminine” and “come to believe that they are the stereotypes.” To buffer this negative influence from the media, Ekow suggested that young Black people, and particularly women, require resilience and a supportive community to bolster their identity in order to feel beautiful and maintain high self-esteem. His comments echo those of an earlier text from Collins, published almost 30 years ago (1986, p. S19), who argued that “[e]nduring the frequent assaults of controlling images requires considerable inner strength.”

Further, several men in our study also emphasised the role the media, or more specifically White people who are in positions of power in the media, plays in placing this beauty burden on Black women. For example, Malakai, a Millennial with dark skin, said:

*[Y]ou are bombarded with these lighter-skin women who are [positioned] as attractive. […] It’s people like Beyoncé, you know, who they believe is beautiful. The Eurocentric blonde hair or the long straight hair, rather than our own hair [which is] considered bad hair. You do not like your kinks in your hair, you think it’s wrong. What’s wrong with it? It’s what grows out of your head. This that you put on is a fake, it’s been placed in your head that that’s what’s attractive. It’s not you who says that’s attractive. It’s what the media, or it’s what the White man, or 400 years of slavery told you that’s attractive: The blue eyes, the lighter skin, the hair.*

Relatedly, Bilal, a Millennial, who had a similar skin shade to Kelly, said:

*If you are looking at the mainstream media that is generally run by White people, now who they consider to be beautiful amongst Black people, and Black people are looking at that, which is generally like light skin or Mixed Race women, that’s why it affects not only Black men, but Black women, and their perception of beauty.*

In their narratives, Malakai and Bilal argued that the media shapes societal perceptions of Black beauty because those in positions of power are White and favour light skin and straight, smooth hair. Finally, Ekow, also a Millennial, argued that even when the media includes Black women, it prioritises those with features that can be associated with whiteness. Using the example of the Black supermodel, Naomi Campbell, he said:

*[E]ven with Naomi, you will not see Naomi with natural hair. So, if you are a young Black girl, and you are looking at that image, or if you are a young Black man and you are looking at that image of Naomi, you are still going to associate how she looks and her features with that of a White woman, or a Eurocentric idea of yes, she’s Black, but she looks closer to being our ideal of what we would like to be Black.*

The implication of Ekow’s argument is that colourism in the media is apparent even when women with darker skin shades are depicted because beyond their skin shade, it is those who fit White appearance ideals in other ways (e.g., thin, straight hair, a slim nose) who are included. This is consistent with Laybourn (2018, p. 2086), who argued that White appearance ideals are maintained by “placing white beauty ideals onto black bodies.” Moreover, his comments resonate with historical perspectives in the media, such as the 1976 description of the supermodel Iman as “a White woman dipped in chocolate” from then editor-in-chief of Essence, Marcia Gillespie (Oliver, 2015).

### 3.2 “The only dark-skin people we like are the dark-skin men”: colourism shaping women’s experiences of romantic relationships

We begin this section with an exploration of how, due to gendered colourism, Black men with dark skin are sought after in the relationship market, while women with dark skin are not. We suggest that Black men’s desirability means that those who have internalised colourism are well-positioned to perpetuate it in their relationship choices. We then complicate this by discussing how men can feel constrained in terms of their dating options due to concerns about other people’s colourist judgements. We conclude the section with an exploration of the issues that sexual colourism raises, including: the pain it can cause Black women with dark skin and the discomfort it can cause those with light skin.

Participants frequently discussed in-group colourism, giving examples of Black people’s prejudicial attitudes and opinions about other Black people based on skin shade. This most commonly arose when speaking about heterosexual relationships where Black and mixed Black-White women with light skin were positioned as the most desirable by Black men, while Black women with dark skin were often overlooked. In contrast, Black men with dark skin were viewed as desirable. This phenomenon can be conceptualised as sexual colourism, drawing on the concept of “sexual racism” (for example, Callander et al., 2015), in which people discriminate between possible romantic or sexual partners based on skin shade.

Sofia and Bilal were among the participants who highlighted gendered in-group colourism, arguing that Black women with dark skin are viewed negatively, while Black men with dark skin are desired. Sofia, who belonged to Generation Z and described her skin as “light golden,” explained:

*A lot of our self-hatred in the Black community comes from Black people. It’s kind of like a mini racism within the Black community. They love the light-skin people, but did not like the dark-skin people. The only dark-skin people we like are the dark-skin men. The dark-skin females, they are seen as aggressive, they are seen as this and that and they are ratchet, whereas the dark-skin men, they are seen as, oh they are the things to have, you know, yeah. And they get cocky over it sometimes, you get a lot of dark-skin men that will trash a dark-skinned woman that’s got the same exact skin tone as him. […] And their mum is the exact skin [as] him, but he’s trashing every Black woman.*

In contrast to the association between femininity and light skin (Hunter, 2002), Sofia suggested that women with dark skin are seen as “aggressive” by other Black people, which is a long-established trope. Assertive Black women are labelled “aggressive” and seen as “threatening” as they “challenge White patriarchal definitions of femininity” (Collins, 1986, p. S17). While Black men with dark skin can be seen as aggressive and threatening too, they are also desired (Phoenix and Craddock, 2022) and Sofia said, they are perceived to be “the things to have.” In commenting that many Black men will disparage (“trash”) women of the same skin shade as themselves and their mothers, she highlights how deeply entrenched gendered colourism can be.

Like Sofia, Bilal described how Black men’s experiences are distinct from women’s because they are sought after in the relationship market. He said:

*Even in a racist society, a Black man is still seen as desirable amongst other races of women, so it does not affect him in terms of the dating pool. […In contrast,] Black women, unfortunately, they suffer from racism, sexism and colourism. They see all these different types of oppression.*

As Robinson (2008) argues, in terms of heterosexual relationships, Black men benefit from more options for relationships with people of other ethnicities than Black women do. For Black women with dark skin, colourism compounds the ‘gendered racism’ or ‘racialised sexism’ to which they are subjected (see Essed, 2013; Kuo, 2017).

The desirability of Black men with dark skin means that those who internalise colourist, gendered appearance ideals are likely to be well positioned to be able to perpetuate it in the relationship market. Using the example of his Black heterosexual male friends’ colourist dating preferences, Ekow said:

*Because their preference is always to go out with White women, and to me that’s on a subconscious level, you have been programmed to think that there’s something wrong with Black women. You’ve all got Black mothers, you all know Black women, you all know beautiful Black women. But you would never want to go out with a Black woman. Or if they do go out with Black women, it will be a very light-skinned Black woman, or a Mixed Race Black woman.*

Through stating that “on a subconscious level” Black men have been “programmed” to view Black women, and particularly Black women with darker skin, negatively, Ekow’s comment emphasises how colourist, gendered appearance ideals can be internalised. The fact that both Sofia and Ekow argue that some Black men shun women with similar skin shades to their mothers, emphasises both the complexity and perniciousness of internalised colourism (Phoenix and Craddock, 2022).

Several Black men in our study provided further nuance to how colourism shapes Black men’s dating preferences by highlighting the impact of concerns that other people might be colourist. For example, Isaiah, a Millennial who described his skin shade as light brown, said he was single and that he would “subconsciously be drawn towards females that are of a similar shade to myself.” When discussing appearance ideals for women, he said, “the ideal has a lot of external factors involved in it.” While his ideal woman would have very dark skin, societal racism and colourism mean that having a partner with dark skin would make him “uncomfortable” and self-conscious when out in public. He gave a hypothetical example:

*So […] we are at a restaurant and the person [waiter] is making only eye contact with myself, not engaging at all with my partner, who’s darker skinned than me, straightaway that would start to make me feel a bit uncomfortable in the sense that I would automatically start thinking it’s because she’s darker as well, but also, other factors, such as she’s a female, and I also subscribe to we live in a patriarchal society. So, it’s almost a double whammy, she’s darker, and she’s a female. And that would make me feel uncomfortable, because I would feel that is unjust.*

His concern that a partner experiencing colourism would make him “uncomfortable” due to the injustice of it, meant that Isaiah said he would choose women with light skin instead. This suggests that societal colourism impacts men in complex ways, affecting not only who they desire, but who they are comfortable to be seen with. Relatedly, Ekow said that his first girlfriend was from South Sudan, and had dark skin, which used to be his preference, and “I knew some people that would make fun of that, like that, because she was dark skinned.” He said that now, “if I see someone that I like, I will ask myself, do I like this person just because they are light skinned? Or am I giving them more preference because they are light skinned? Because I’m aware that that happens.” This raises the question of whether his change in preference was related to the mocking he experienced while in his first relationship. These narratives suggest that concerns about the implications, and potential stigma, of being seen with women with dark skin can inform Black men’s dating preferences.

Some participants also described how such in-group sexual colourism created tensions between Black people and was particularly harmful to Black women with darker skin shades. For example, Erika, a Millennial who said her skin was dark brown, described feeling pain and marginalisation due to experiencing colourism from men with dark skin. She said:

*[T]he ones who held the strongest kind of opinions about light skin being the right skin, were dark-skin men. So, at the time that was a little painful because it was men who look more like you, than anybody else. So, I do not really know. I did not feel pressure, but I kind of felt like marginalised, or at least like they were my type, so, it felt like you were not your type’s type. So, where do you kind of go from there? Because they were very myopic in that view, it wasn’t like it could change and it wasn’t that they would not find darker-skin girls attractive, it was just that they would not consider it. It would just be, she needs to be light skin, she needs to have green eyes.*

Her comment that “it felt like you were not your type’s type. So, where do you kind of go from there?” conveys a concern that Black women with darker skin frequently express (for example, see McClinton, 2019), namely the idea that if Black men with dark skin shun Black women of the same skin shade when it comes to dating and relationships, Black women with darker skin are overlooked in the relationship market. Erika points out that it is women with light skin, “the right kind of hair” and green or light eyes that are desired, rather than women who look like her.

In contrast, Jennifer, a Millennial who described her skin as “tanned,” said she was desired by Black young men, but their preference for women with light skin made her feel “uncomfortable.”

*[Y]ou hear people say, ‘Ooh, I like –,’ which it just makes me so uncomfortable but – ‘Oh, I like light-skinned girls.’ That is like a huge thing for like young people, or like young Black men. And it does not sit well with me because then it’s almost like I’m privileged in some way, even though I do not particularly feel like I am. Or people say, ‘Oh like – you are like the best of both’ […] like ‘the perfect mix of White and Black’ […and] people think you should take that as a compliment, but I do not think that […] is really a compliment, you are actually, again, just being like very ignorant. But I definitely think that people think that I’m more desirable, especially to Black boys because I’m Mixed Race and that’s what they like prefer.*

Rather than enjoying being privileged because of her light skin, she said she did not feel particularly privileged and found the way her skin shade and mixedness were lauded by others as special to be “ignorant.” Jennifer was not alone in considering that light skin is not always a privilege. Some of the mixed Black-White participants said they were fetizishized and excluded from belonging to an imagined community of Black people, which we discuss in detail elsewhere (Phoenix and Craddock, 2024b).

### 3.3 “Competitiveness between skin shades”: the pernicious impact of colourism on relationships between women

In this section, we explore how the inequalities that colourism engendered led to tensions between Black women with dark skin and Black and mixed Black-White women with lighter skin shades. We begin by examining perceptions that women with light skin consider themselves superior to their counterparts with darker skin. We then analyse narratives that argue that sexual colourism leads to competition between women with dark skin and those with light skin, who are privileged in the relationship market. We conclude by suggesting that tensions between women might be exacerbated due to the perception that mixed Black-White women downplay colourism.

Some participants described how Black and mixed Black-White women with light skin were stereotyped as being superior and argued that this harmed relations between women of different skin, a finding that resonates with Spratt’s (2024) study. She found that Black women with dark skin argued that women with light skin felt that they were “special” and might avoid engaging with Black women with dark skin, preferring the company of White women and those with light skin, whom they considered their equals. Similarly, in our study, Erika suggested that women who were Mixed and/or had light skin felt superior, which led to tensions:

*There was a superiority complex that came from it, knowing that you were kind of the most desired and sometimes it got tense […]. I did not feel girls [were] being like “I’m the best,” it was more like “I know they see me as the best.”*

In parallel to this, women in the study with lighter skin described how they perceived they were viewed by Black women with darker skin. For example, Jennifer shared her experience as a woman with light skin who faced “resentment” from Black women with darker skin who assumed she was conceited because her skin shade made her desirable.

*I’m like, light skinned, which is what the boys say that they like, they like light-skinned girls and I think– I think people automatically assume that you have that confidence, that that would make me super confident and that I’m aware that boys like me, so they automatically kind of think that you are a bit up yourself, even though I’m like very far from being up myself. I think people think that I must know that people think I’m like attractive.*

Jennifer argued that in contrast to misconceptions about her that relate to stereotypes about women with light skin (“people automatically assume that you have that confidence”), she is “very far from being up myself,” (i.e., being conceited or arrogant). Similarly, Sofia, who also had light skin, suggested that women with skin shades similar to hers are resented due to the light-skin privilege that some benefit from and perceptions that they are “stuck up” and conceited, and “not all that” (i.e., not as special as they think they are).

*You also get this myth that dark-skin girls are jealous of light-skin girls too, so that also plays a part in it because then you get automatically stereotyped as being like bougie. Light-skin girls always get that stereotype that we are stuck up, we are bougie, we think we are pretty […]. And dark-skin girls like, they just think we are not all that. […] stereotypes hinder stuff a lot.*

Notably, participants in our study described how these stereotypes were not benign. For example, for Sofia, stereotypes about Black women with light skin impeded her ability to have relationships with Black women with dark skin. She said:

*I am more friends with light-skin people than dark skin, and not on purpose. Like, I would like to become friends with more dark-skinned people, it’s just like, well, they would not be friends with me.*

Sofia’s comments highlight the barriers that colourism, and the resultant mistrust between women with dark and light skin, can create. Her narratives suggest that from her perspective, her skin shade is used to stereotype her, and she is seen as a type, rather than an individual in her own right.

Furthermore, several of the mixed Black-White women participants from different generational cohorts said that they faced hostility from Black women with darker skin due to competition in the relationship market and resentment about their light-skin privilege. For example, Ella, who was Generation Z and described her skin shade as a “yellowy colour,” said that on social media women with light skin were constructed as denigrating Black women and “stealing” Black men:

*There’s a lot of posts on Instagram about lighter skin women being, like either stealing Black men or being, putting Black women down. Things like thinking that they are better because their skin is lighter, and I do feel like sometimes that Black women treat me differently for those reasons. Because maybe they have a perception of me, like I’m going to be stuck up or think that I’m better than them.*

Ella suggested that Black women treat her “differently” because they think she feels superior due to her light skin. The narratives in the previous section about Black men, including those with dark skin, shunning Black women and desiring Mixed women and those with light skin, help to contextualise what Ella describes as online posts about women with light skin “stealing” Black men.

Similarly, Grace, who was Generation X and described her skin as ‘dark for someone who was Mixed’, was more explicit about the way in which she feels Black women treat her. She said:

*I definitely felt like I got microaggressions and passive aggressiveness from Black women. Especially if I was seen out with a Black man, I was given the impression sometimes that, you know, it’s not my business to be dating a Black man. Also, when you are out at clubs and stuff, and I would predominantly only go to Black spaces, I would feel most comfortable there. […] But I would usually get vibes from the women, not from the men, that I should not be there. And I think that’s cause of the competitiveness between skin shades and I’ve always felt that and even now […] there’s still [a] feeling of you should not be here.*

Grace argued that Black women subjected her to “microaggressions” and “passive aggressiveness” and made her feel that she should not date Black men or frequent what she described as “Black spaces” due to “the competitiveness between skin shades.” In doing so, she seemed to be describing hostility driven by colourism-fuelled competition, and particularly competition for Black men, between Black and Mixed women, or those with dark skin and those with lighter skin shades.

Jennifer, a Millennial, made similar points, arguing that she feels drawn into competition with Black young women who feel she is encroaching on their space, when she does not wish to compete. Her comments resonate with Campion’s (2019, p. 200) argument that Black and mixed Black-White women “can, unwillingly, find themselves pitted against other women in competition for male approval.” Jennifer said:

*I’ve definitely, definitely been in situations before, where there’s been a group of Black girls and they have kind of been giving me the side eye, like, ‘Who do you think you are?’ Kind of thing, which again it’s like, I’ve been thrown into a competition that I never wanted to be in. They think that we are like there to compete with each other, whereas I’m literally not interested at all, but I definitely think there’s like a competition thing and I think because I’m not Black, I’m Mixed Race, it’s – I almost feel like…they think that I’m like stepping on their territory and I do not deserve – like I do not deserve to be like in that because I’m not Black.*

Her description of Black young women giving her “the side eye” suggests that she faces microaggressions in a similar way to Grace. Despite stating that she does not want to be in competition with Black women, the fact that she has tanned skin and is Mixed ethnicity, means that she has an advantage in the relationship market over Black women who may consider her their competitor.

Further complicating relationships between Black women with dark skin and Black and mixed Black-White women with light skin, some participants with darker skin shades argued that those with lighter skin often downplay colourism, and their perspectives tend to be prioritised over those of Black women who experience the negative effects of the prejudice. For example, Bilal said:

*[T]here is a reluctance, even when the issue of colourism comes up, there’s a lot of pushback from a lot of Mixed Race and light-skinned Black women because they all jump on it, “We’re all the same, it does not matter,” which we are, but there is clearly a hierarchy, unfortunately, which exists among a lot of men, in terms of when they are viewing Black women. When you have got a group of Black women speaking about it and then the Black men are denying it and you have got Mixed Race and light-skin women who say, “No it’s not true, it’s exaggerated,” that’s the reason why a number of Black men would not even listen to Black women, because you have got other women who are saying, “It does not exist, it’s just in their heads,” so to speak.*

Bilal suggests that Black men choose to listen to women of Mixed ethnicity and those with light skin who argue that colourism claims are “exaggerated” and only in Black women’s heads, rather than listening to the perspectives of Black women with darker skin, who are lower down the hierarchy. While he does not discuss the impact of this on relations between women of different shades, from the preceding narratives it seems likely that such scenarios would cause frustration and potentially resentment.

In a similar way, Erika said “a lot of the time it’s denied,” with people declaring that they just have a particular preference. As Silvestrini (2020, p. 308) argues, sexual colourism “reproduces hierarches of race and power under the illusion of personal preferences towards racial ‘types’.” Erika described the work she does to try and get people to understand that if everyone has the same narrow preference then something more is going on. However, she suggested that people can perceive her to be “bitter” or an “angry Black woman” for bringing up the topic.

*It’s like you are being the angry Black woman who’s annoyed that people do not find you attractive […] Dark-skin women cannot accept that light-skin women are more desirable. And it’s like, that is not the issue. Honestly, I do not care. If that is your type, great. But at least come from a place of being informed. Because a lot of the time you have not even, you are myopic, you cannot see beyond, a lot of the time, what you have been told to like. Because you have not even thought about why you like this, what the odds are, what the chances. It’s very much a cookie cutter. Everybody likes this, so you should too.*

Erika suggested that women with dark skin are seen as jealous (“Dark-skin women cannot accept that light-skin women are more desirable”). In doing so, she both highlighted tensions between women of different shades, as discussed above, and underlined how difficult it is to challenge colourism among Black people when the views of those critiquing the prejudice are trivialised.

### 3.4 Small acts of resistance: rejecting colourist ideology and embracing Black beauty

This final section focuses on participants’ narratives about countering colourism in the context of gendered appearance ideals and self-worth. Accordingly, we examine how participants discussed the need to resist colourist appearance ideals through different strategies including critiquing and rejecting societal appearance ideals, education, and taking pride in their skin shade and appearance.

Some participants emphasised the importance of rejecting racist and colourist ideologies that tell Black people with dark skin that they are inferior and ugly. Malakai, for example, emphasised the need critically to appraise appearance ideals and to recognise the contradictions inherent in the fact that physical features that are associated with, but not privileged in, Black bodies, are increasingly celebrated in White bodies. Implicitly, Malakai called for Black people to reject the dominant racist and colourist messages. He said:

*[Y]ou look at the Kardashians now, filling their lips to get bigger lips, fuller lips, what you already have, bigger bums, what you already have, this curvier body, what you already have, but they told you that wasn’t attractive, but now they are taking it for themselves. They love the culture, they do not like you, and they make you feel worthless while they are stealing what you have. Look at it differently. Think about it.*

Malakai suggests that mainstream appearance ideals are racist and colourist with White women desiring (and having surgery to achieve) features that are denigrated when associated with Black women. Although he does not use the term, what he is describing is a phenomenon known as “blackfishing,” which includes “altering physical appearances through physical and digital means” to appropriate black aesthetics (Stevens, 2021, p. 1). When he says, “[T]hey make you feel worthless while they are stealing what you have. Look at it differently. Think about it,” he invites an imagined audience of Black women to reflect on the injustice and contradictions inherent in appearance ideals. “Look at it differently” may be a call for Black women to reevaluate both their perceptions of beauty and their self-perceptions.

Just as Malakai called for Black women to reflect and change their perspectives, Portia, who was in an older generational cohort, described her ‘journey’ of “re-educat[ing]” and “de-brainwash[ing]” herself.

*[Y]oung people use kind of language around people being ‘blick’ and you know, I hear things all the time that kind of make you think, wow. I do not know, are we really moving beyond this state that we were in, or are we standing still? I’m not sure. For me personally, I’ve moved a long way along that journey, I’m definitely in a better place now. But that’s through educating myself, re-educating myself, de-brainwashing myself, going into spaces where they are all Black people in that space, and no one seems particularly– well on the surface they do not seem bothered with what shade you are.*

Portia argued that colourism is still prevalent, and it is not clear that progress is being made at a collective level, though she has educated herself and worked on addressing her internalised colourism in a way that arguably enable her to “[l]ook at it differently.” As Glenn (2008, p. 298) argued, attempts to address colourism are often individualised and framed around “re-education” that focuses on broadening conceptions of beauty and desirability to include dark skin so that whiteness and light skin are no longer viewed as the “dominant standard.”

Other women focused on taking pride in the darkness of their skin and the way that they looked in a moment when there is a renewed move to celebrate natural Black beauty. For Kelly, ensuring she was “presentable” was an important way to challenge negative colourist narratives about Black women with dark skin, demonstrating that “we are beautiful as well.” Her comments evoke the “Black is Beautiful” slogan from the 1960s and 1970s Black Consciousness and Black Power movements, which protested against institutional racism and sought to celebrate Black features, including dark skin, Afro hair and Black cultures (Baird, 2021). Kelly said:

*I like to, for most of the time, be presentable and…this might sound really funny but represent in a way, because I think there’s so much sort of negative viewpoint when it comes to darker skin and Black women, that I think it’s important to show, do you know what, this is us and actually we are beautiful as well. So, I tend to really take pride in my appearance in certain environments.*

Kelly struck a defiant tone as she argued that she took pride in her appearance to emphasise the beauty in women with dark skin that mainstream colourist narratives seek to deny. Collins (1986, p. S24) asserts that when Black women draw on whatever is at their disposal “to be self-defined and self-valuating [*sic*] and to encourage others to reject objectification, then Black women’s everyday behaviour itself is a form of activism.” She suggested that just the act of viewing oneself “as fully human, as subjects,” is enough to become an activist even if only in a limited way. In her narrative, Kelly rejects definitions of Black women with dark skin as ugly or unattractive and asserts her own “definition” of Black women with dark skin as beautiful, which could be seen as a form of resistance or “activism.”

When asked how she felt about her skin shade, Destiny said, “I love it, I always have.” She said, “I was lucky growing up in somewhere like [area in London] where everyone was from a different place […]. [I]n places like [area in London], Blackness is celebrated, so I was really happy.” Her appreciation for her skin shade was reflected in her description of how she puts on makeup:

*[A]nd then I put a bit of bronzer on, and I like the one that’s quite brown and full, it just looks better with my face. Rather than if I wanted to make it lighter, I prefer to go darker, I think it looks better.*

In a similar way to Kelly, Destiny took pride in her appearance and presented herself to the world in a way that emphasised the fact that she saw darker skin as valuable.

Ekow pointed to changes in perceptions around beauty, suggesting that there has been a shift in how Black people both see and accept themselves:

*There’s a lot more men and women embracing their natural hair, and embracing themselves, and are being able to articulate more now, that growing up, they did not feel worthy, they did not feel beautiful, they did not feel handsome. But they now are beginning to feel and accept themselves a lot more.*

He contrasts Black people’s comments about feeling inferior and unattractive as they were coming of age, with what he perceives to be them “beginning to feel and accept themselves” much more. His comments should be contextualised against a backdrop of digital and hybrid movements that centre on celebrating black beauty and natural hair. Such movements include the ‘natural hair’ movement and the “#BlackGirlMagic” hashtag, which “insists on the visibility of black women and girls as beauty subjects and aspirational figures in the wider culture” in an effort to challenge discourses that construct Black women as dysfunctional, unattractive and “social failures” (Hobson, 2021, p. 4).

## 4 Discussion

In this paper, we examined the complex processes through which colourism situates Black and mixed Black-White women in contrasting positions in beauty and desirability hierarchies based on their skin shade. We took an intersectional approach to our analysis to allow us to be particularly attentive to participants’ positionality in terms of gender, skin shade, and where possible, their generational cohort. Our findings contribute to international work on colourism by presenting a British perspective, and providing an in-depth account of colourism’s role in the heterosexual relationship market, and the effect of sexual colourism on platonic relationships between Black and mixed Black-White women.

Women and men in our study argued that gendered colourism in the media, and society more broadly, could be internalised by Black people, leading both to negative self-perceptions among women with dark skin, and negative perceptions of women with darker skin shades. This supports US qualitative research with Black women and adolescent Black girls that emphasises the negative impact of gendered colourism on participants (Hall, 2017; Abrams et al., 2020). Triangulating our findings with those in the US with different age groups underscores the similarity of experiences of Black girls and women across the Atlantic.

Notably, alongside a lack of representation of women with dark skin, participants in our study often highlighted the lack of representation of Afro hair in media portrayals of female beauty. This is consistent with work by Joseph-Salisbury and Connelly (2018), who argue that Black hair practices need to be contextualised within a racialised social structure that privileges White appearance ideals and stigmatises physical features associated with Black people, such as skin colour and hair. Together, this highlights the need to consider hair texture, alongside skin shade, in analyses of how gendered colourism affects Black women.

Women and men in our study also described how gendered colourism, perpetuated by appearance ideals in the media, is internalised and enacted by Black men, informing their (heterosexual) relationship choices. Specifically, Black men rejected Black women with dark skin in the heterosexual relationship market, preferring Black women with light skin or White women. Participants described how this led to competition between Black women with dark skin and Black and mixed Black-White women with light skin. This is consistent with Campion (2019), who asserted that Black and mixed Black-White women can be drawn into competition for male attention. Campion argued that “the systematic racism, sexism and colourism experienced by darker skinned Black women can create frustrations which, in some cases, can result in ‘interpersonal conflict’ with Black mixed race women.”

Some of our participants described small acts of resistance whereby Black people, including those with dark skin, rejected colourist ideology and embraced notions of Black beauty. While this was presented as a sign of hope in the face of colourist controlling images, individualised rejection of colourism is insufficient to combat the prejudice. What is required is the dismantling of the structures that sustain racism and colourism. As Glenn (2008, p. 298) argues, “focusing only on individual consciousness and motives distracts attention from the very powerful economic forces that help to create the yearning for lightness and that offer to fulfil the yearning at a steep price.” Multinational corporations profit from the lucrative global skin lightening industry and are therefore invested in perpetuating colourism in order to create and sustain demand for products that depend on inequities and insecurities created by colourism (Glenn, 2008). Addressing colourism will require educating and regulating both the corporations that profit from it and the institutions that help to sustain it (cf. Hill, 2002; cf. Phoenix, 2014).

### 4.1 Limitations

There are three key limitations of the study worth consideration. First, we employed convenience sampling, and our resulting sample included almost twice as many Black or mixed Black-White women with light skin as Black women with darker skin shades. This means that we have an imbalance in the sample that may have skewed the data on perceptions of relationships between women with dark and light skin and how they are affected by colourism. Relatedly, fewer men participated than women, meaning that we had fewer male perspectives to draw on. Second, our findings may be affected by a selection bias based on our recruitment strategy in which minoritised ethnic people were invited to take part in an interview study about “skin shade.” We may, therefore, have attracted those for whom skin shade is particularly salient and who would be happy to talk about the role of skin shade in their lives.

Third, it would have been valuable to include a specific question on relationships with women of different skin shades to capture more perspectives on how colourism affects interpersonal dynamics between Black and mixed Black-White women with different skin shades. However, the fact that many participants commented on this despite not being asked directly, could be viewed as a strength of this inductive analytical work. Finally, though we collected age data, the spread of participants’ ages in conjunction with our relatively small sample size meant that we were often unable to make meaningful comparisons or interpretations by age.

### 4.2 Future directions

Colourism research is still relatively nascent in the UK. Therefore, there are several directions of research that could build upon the current work focused on colourism and romantic relationships in the UK. First, it would be valuable to conduct a larger in-depth UK study with Black and mixed Black-White women, capturing the perspectives of more women with dark skin and specifically asking women of all shades how they perceive and relate to Black and Mixed women of different shades, as well as asking them about their desire and experiences in the relationship market. Second, it would be beneficial to conduct similar research on colourism and relationships with minoritised ethnic people of other ethnicities in the UK. Third, understandings of colourism in the context of relationships would benefit from more research with minoritised ethnic people who occupy marginalised identities related to sexual orientation and gender to expand intersectional analyses. Fourth, more nuanced intersectional understandings of colourism and relationships could be gained by considering personal characteristics such as social class and generation status.

### 4.3 Conclusion

This study highlights how colourist and gendered appearance ideals can affect Black and mixed Black-White women’s experiences in the heterosexual relationship market. We argued that Black women with dark skin and Afro hair were seen as unattractive due to sexual colourism, in contrast to the perceived beauty of mixed Black-White women, who were desired and sometimes objectified. Participants argued that dominant media constructions of white and light skin as beautiful and desirable, and dark skin as the opposite, could lead Black women with dark skin to internalise the idea that they are ugly, while Black men shunned them in favour of women with lighter skin in the romantic relationship market.

We found that sexual colourism damaged platonic relationships between women of different shades, some of whom felt they were thrust into competition with each other, creating resentment and mistrust. In this way, the study reveals some of the subtle, less tangible effects of colourist, gendered appearance ideals, which shape Black and mixed Black-White women’s realities. Our intersectional approach to analysing how sexual colourism affects Black and mixed Black-White people, and particularly women, was valuable as it enabled us to be attentive to the complex ways in which intersecting factors such as gender, racialisation and skin shade affected both our participants’ experiences and the possibility of heterosexual and platonic relationships. In particular, it allowed us to highlight the detrimental impact of sexual colourism on Black women with dark skin.

## Data Availability

The datasets for this article are not publicly available as participants have not consented to the archiving of their data. Requests to access the datasets should be directed to the corresponding author.

## Footnotes

^1^We acknowledge that the term ‘People of Colour’ is imperfect. It implies that White people have no colour and thereby renders whiteness invisible. Where possible we have specified particular ethnic/racialised groups, but occasionally for simplicity and brevity, a collective term is useful. Mostly, we use “minoritised ethnic people,” but where a broader term is required, we use ‘People of Colour’.

^2^Anti-black racism is defined as a “system of beliefs and practices that attack, erode, and limit the humanity of Black people” (Carruthers, 2018, p. 26).

^3^Mixed Black-White ethnicity data is not available for Scotland, though the total Mixed Ethnic Group category for Scotland is 1.1%.

^4^While the study was open to people living in the UK, only people living in England and Scotland responded, thus Britain better represents the study sample compared with the UK.
